# Refining the Role of Tumor-Associated Macrophages in Oral Squamous Cell Carcinoma

**DOI:** 10.3390/cancers17172770

**Published:** 2025-08-25

**Authors:** Kiyofumi Takabatake, Piao Tianyan, Takuma Arashima, Anqi Chang, Hotaka Kawai, Htoo Shwe Eain, Yamin Soe, Zin Zin Min, Masae Fujii, Keisuke Nakano, Hitoshi Nagatsuka

**Affiliations:** Department of Oral Pathology and Medicine, Graduate School of Medicine, Dentistry and Pharmaceutical Sciences, Okayama University, 2-5-1 Shikata-cho, Kita-ku, Okayama 700-8525, Japan; pfd12zwy@s.okayama-u.ac.jp (P.T.); de428001@s.okayama-u.ac.jp (T.A.); pmff7em4@s.okayama-u.ac.jp (A.C.); de18018@s.okayama-u.ac.jp (H.K.); pmp61kpp@s.okayama-u.ac.jp (H.S.E.); pki31ld5@s.okayama-u.ac.jp (Y.S.); pobk1heq@s.okayama-u.ac.jp (Z.Z.M.); gmd20089@s.okayama-u.ac.jp (M.F.); pir19btp@okayama-u.ac.jp (K.N.); jin@okayama-u.ac.jp (H.N.)

**Keywords:** tumor-associated macrophage (TAM), oral squamous cell carcinoma (OSCC), macrophage polarity, invasion, carcinogenesis

## Abstract

In the tumor microenvironment, various immune and stromal cells, such as fibroblasts and vascular endothelial cells, contribute to tumor growth and progression by interacting with cancer cells. Tumor-associated macrophages (TAMs) have attracted attention as major players in the tumor microenvironment. The origin of TAMs is believed to be the infiltration of monocytes derived from bone marrow progenitor cells into tumor tissues and their differentiation into macrophages, whereas tissue-resident macrophages derived from yolk sacs have recently been reported. TAMs infiltrating tumor tissues act in a tumor-promoting manner through immunosuppression, angiogenesis, and the promotion of cancer cell invasion. Reflecting the nature of TAMs, increased TAM invasion and TAM-specific gene expression in tumor tissues may be the new biomarkers for cancer. Moreover, new therapeutic strategies targeting TAMs, such as transformation into immunostimulatory macrophages, suppression of TAM infiltration, and promotion of phagocytosis, are being investigated, and many clinical trials are underway. As the origin and function of TAMs are further elucidated, TAM-targeted therapy is expected to become a new option for the immunotherapy of various cancers, including oral cancers.

## Simple Summary

Oral squamous cell carcinoma (OSCC) is a highly invasive cancer influenced by the tumor microenvironment. Tumor-associated macrophages (TAMs) play key roles in promoting OSCC progression through interactions with cancer cells. TAMs support tumor progression by suppressing immune responses and enhancing angiogenesis. Recent studies highlight the complex crosstalk between OSCC cells and TAMs, which influences tumor behavior and patient prognosis. Understanding these interactions may lead to novel therapeutic strategies targeting the tumor microenvironment. This review summarizes the latest findings on the roles of TAMs in OSCC, emphasizing their potential as therapeutic targets for improving patient outcomes.

## 1. Introduction

Head and neck cancer ranks sixth among all malignancies, with approximately 550,000 new cases diagnosed annually worldwide. Of all head and neck cancers, oral cavity cancers affect the largest number of people, with approximately 37,700 new cases diagnosed and 17,800 deaths worldwide [1]. Currently, surgery is the first choice of treatment for most oral squamous cell carcinoma (OSCC) cases, sometimes in combination with radiation therapy, chemotherapy, or immunotherapy [2]. Despite advances in the understanding of OSCC biology, the patient survival rate after 5 years is approximately 50%, mainly due to the presence of regional lymph node metastasis [3].

One of the factors that make the treatment of OSCC challenging is the presence of a tumor microenvironment (TME). In the TME, stromal cells other than cancer cells, such as various immune cells, fibroblasts, and vascular endothelial cells, contribute to cancer cell growth and progression by interacting with cancer cells [4,5]. Tumor-associated macrophages (TAMs) are major components of tumor stromal cells that promote tumor growth by promoting cancer cell proliferation, invasion, angiogenesis, and immunosuppression. Therefore, they are attracting attention as new biomarkers for tumors, and the possibility of new cancer therapies through their regulation is being explored. This article outlines the origin of TAMs in the oral squamous cell carcinoma tumor microenvironment, the relationship between TAMs and carcinogenesis, and that between TAMs and tumor invasion based on recent research findings; moreover, it discusses future research directions and potential therapeutic applications.

## 2. Methods

The search was performed on PubMed, SCOPUS, and Web of Science with the following terms: “oral squamous cell carcinoma”, “macrophage”, “tumor associated macrophage”, and “TAMs” (last access in February 2025). Studies were included if they met the following criteria: full-text availability, publication in English, and direct relevance to the origin of TAMs, the polarization of macrophages in tumors, carcinogenesis of oral squamous cell carcinoma and tumor invasion associated with TAM, and biomarker of TAMs.

## 3. Origin of TAMs

**Figure 1:**
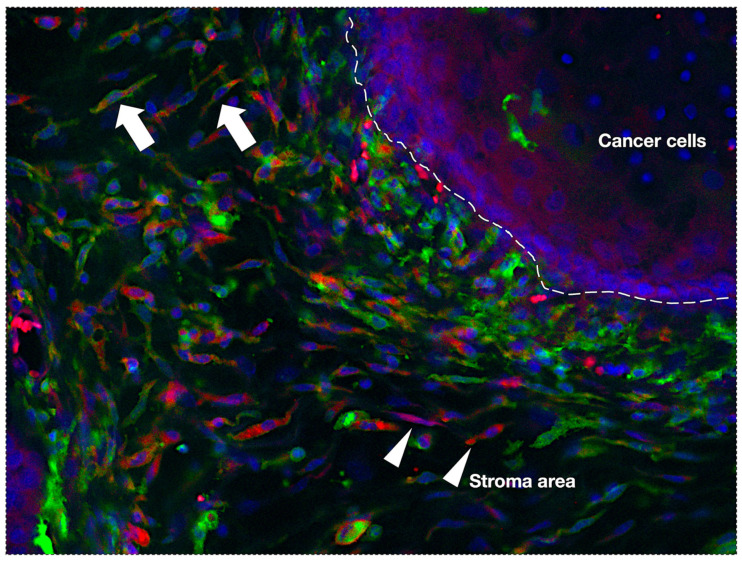
Immunofluorescent double staining image of GFP-positive bone marrow-derived cell transplantation OSCC mouse model. Most of the F4/80-positive cells merge with GFP-positive cells (arrows), suggesting that most of the TAMs are derived from bone marrow. However, some F4/80 alone-positive cells (arrow heads) are observed, suggesting the presence of yolk sac-derived TAMs in the cancer stromal area. Magnification ×100. Green color; anti-GFP antibody, Abcam, Boston, MA, USA, ab6673, 1:100; red color; anti-F4/80 antibody, BIO RAD, Hercules, CA, USA, C1:A3-1, 1:10.

Since the seminal work by van Furth et al. in 1968, it has been widely accepted that macrophages, including tumor-associated macrophages (TAMs), primarily originate from peripheral blood monocytes derived from bone marrow progenitor cells [6]. However, in the early 2000s, studies revealed the existence of tissue-resident macrophages that arise independently from the bone marrow, instead originating from yolk sac progenitors during embryogenesis [7]. Bone marrow-derived TAMs are thought to be derived from peripheral blood monocytes that migrate to tumor tissues and differentiate into TAM via chemokine and cytokine signaling produced by tumor and stromal cells. Among these signals, colony-stimulating factor (CSF)-1 plays a central role by acting via the CSF1 receptor on monocytes [8]. Other factors, such as chemokine ligand (CCL)2 (MCP-1), CXC motif chemokine ligand 12, vascular endothelial growth factor (VEGF), and CCL20, have been reported to promote monocyte migration via their respective receptors on monocytes [9,10,11,12]. In contrast, tissue-resident macrophages are thought to originate from yolk sac progenitor cells and differentiate into macrophages upon stimulation by inflammation or tumors [7]. It has recently become clear that cells in various organs, such as microglia in the central nervous system, Kupffer cells in the liver, alveolar macrophages, and Langerhans cells in the skin, are tissue-resident macrophages with a common origin of yolk sac progenitor cells. However, the effect of differences in macrophage origin on macrophage function in the tumor microenvironment remains largely unknown. For instance, in pancreatic ductal adenocarcinoma models, tissue-resident macrophages have been implicated in tumor growth and fibrosis, while bone marrow-derived macrophages appear to exhibit stronger antigen-presenting capabilities [13]. The role of different origins of TAMs in OSCC has not been adequately studied, and even the origin of TAMs has not been adequately reported. Our recent study demonstrated for the first time the infiltration and differentiation of bone marrow-derived macrophages in the oral cancer microenvironment using a GFP-positive bone marrow transplantation mouse model. The results confirmed that most macrophages in the oral squamous cell carcinoma stroma were derived from the bone marrow; however, some non-bone marrow-derived macrophages infiltrated the tumor microenvironment (Figure 1). In the future, because of differences in the origin of macrophages in many malignant tumors, their effects on the function of macrophages in the tumor microenvironment and the mechanism of tumor invasion may become better understood, and the possibility of new cancer therapies through their control is anticipated.

## 4. Polarization of Macrophages

Macrophage polarization is generally divided into two main phenotypes: M1 and M2 [14]. M1 macrophages are activated by the inflammatory cytokines, phagocytosis, and antigen presentation of foreign materials by natural immune responses and are involved in helper T cell (Th) type 1 responses. In addition, Th1-type cytokines, including interferon-γ and interleukin-12, along with external stimuli such as lipopolysaccharides, promote the differentiation of macrophages toward the M1 phenotype. On the other hand, M2 polarization is facilitated by Th2-associated cytokines, such as IL-4, IL-10, and IL-13, which are implicated in anti-inflammatory responses, extracellular matrix remodeling, and the formation of new blood vessels.

Although the polarization state of macrophages is heterogeneous, especially in complex and fluid environments, such as the TME, TAMs often exhibit an M2-like phenotype in many malignant tumors and have been found to act in a tumor-promoting manner [15]. It has been reported that lactic acid produced by tumor cells is mediated by a hypoxia-inducible factor (HIF-1α) and that lactate-induced arginase 1 expression in macrophages induces M2-like polarization of macrophages in a melanoma murine tumor model [16].

In the TME of OSCC, TAMs and cancer cells are in close proximity and close contact, and close cell–cell interactions between TAMs and cancer cells appear to exist. It was recently shown that factors secreted by cancer cells can affect macrophage polarization. Furthermore, recent studies have advanced our understanding of macrophage polarization (M1/M2) in the TME of patients with OSCC. Dan et al. reported that RACK1 expression in OSCC cells has been implicated in promoting TAM accumulation and M2 polarization through NF-κB signaling [17]. Additionally, circ-ILF2 derived from OSCC cells has been found to induce M2 polarization in macrophages [18].

Moreover, OSCC-derived exosomal miR-29a-3p contributes to the polarization of macrophages toward the M2 subtype via activation of the SOCS1/STAT6 signaling pathway in macrophages [19]. Furthermore, CCL19 and CCL19 production driven by CCR7 expression in OSCC cells promotes the recruitment and M2 polarization of THP-1-derived macrophages [20]. Additionally, OSCC cell-secreted exosomal CMTM6 induces M2-like macrophage polarization through the ERK1/2 signaling pathway, thereby promoting malignant progression and highlighting a novel interaction between cancer and immune cells in the OSCC microenvironment [21]. As described above, it has recently been reported that in OSCC, factors secreted by cancer cells promote the accumulation of macrophages in the tumor microenvironment and polarize them to the M2 type. In contrast, exosomal THBS1 secreted from OSCC cells is involved in the polarization of macrophages toward an M1-like phenotype [22].

Conversely, the OSCC tumor stroma influences macrophage aggregation and polarization. We created a mouse model of oral squamous cell carcinoma cell lines implanted with two tumor stroma subtypes and analyzed the aggregation and polarization of macrophages into the tumor tissue. Our results showed that more macrophages infiltrated the TME, and most of them were polarized to the M2 type in mice implanted with cancer stroma established from endophytic OSCCs compared to that derived from exophytic OSCCs [23]. Thus, it is clear that both cancer cells and cancer stromal cells influence macrophage aggregation and polarization in the TME of OSCC.

While the M1/M2 classification has been instrumental in characterizing macrophage functions in infectious and immune-related disorders, growing evidence indicates that tumor-associated macrophages (TAMs) within human solid tumors represent a diverse and heterogeneous population. In the actual TME such as adenocarcinoma and cutaneous SCC, complex immune responses are induced, and macrophages are not simply bipolarized, as described above; macrophages are a mixture of cells in various states of polarization [24,25]. Bill et al. proposed an alternative classification based on CXCL9 and SPP1 (CS) expression, which demonstrated a stronger prognostic correlation in head and neck cancers than traditional M1/M2 markers. Furthermore, CXCL9 and SPP1 expression in macrophage polarization has been shown to orchestrate a spatially structured and tightly regulated network of tumor-promoting and tumor-inhibiting factors, involving diverse populations of tumor-associated cells. Taken together, these findings imply that, although the tumor microenvironment (TME) is highly complex, it can elicit coordinated responses that influence cancer progression, with colony-stimulating factor-mediated macrophage polarization serving as a meaningful yet tractable component [26]. Therefore, instead of conventional polarization using M1 and M2 markers, we investigated the relationship between macrophage-derived humoral factors and OSCC prognosis.

## 5. TAMs and Carcinogenesis

The relationship between chronic inflammation and carcinogenesis has been studied extensively for over a century, dating back to Virchow’s initial observations in the 19th century [27]. TAMs have been shown to play a pivotal role in tumor initiation and progression. In colorectal and hepatocellular carcinomas, TAM-derived IL-6 promotes carcinogenesis via activation of STAT3 signaling [28,29]. Additionally, IL-8 secreted by TAMs has been implicated in STAT3 activation and may be involved in tumorigenesis, whereas IL-17 and IL-23 promote carcinogenesis in colorectal cancer [30]. During tumorigenesis involving macrophage activation, the TME undergoes a shift from a Th1-dominant profile to a Th2-dominant state, facilitating the polarization of macrophages toward the M2 phenotype under the influence of IL-4 and IL-10 secreted by cancer cells.

**Figure 2:**
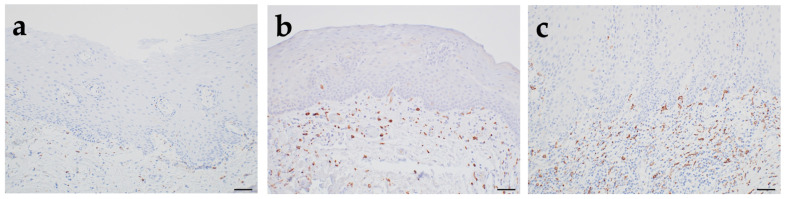
Immunohistochemical staining image showing macrophage aggregation in the surrounding area of normal epithelial (**a**), low-grade epithelial dysplasia (**b**), and high-grade epithelial dysplasia (**c**). Scale bar; 50 μm. Anti-CD163 antibody, abcam, ab182422, 1:200.

Accumulating evidence suggests that macrophages contribute to the progression of epithelial dysplasia toward OSCC. Immunohistochemical analyses have shown that normal oral mucosa lacks significant infiltration by macrophages expressing CD68, CD80, or CD163. However, in moderate epithelial dysplasia, CD163-positive macrophages become more prominent, particularly in periepithelial regions [31]. Our recent histological study demonstrated that in normal oral mucosa, CD163-positive cells are rarely observed. On the other hand, in low-grade epithelial dysplasia, CD163-positive cells with a round shape increase around the epithelial tissue, and in high-grade epithelial dysplasia, CD163-positive cells with a dendritic form cluster more around the epithelial tissue (Figure 2). These findings support the hypothesis that macrophages, particularly the M2 macrophage, may actively participate in the malignant transformation of oral premalignant lesions such as leukoplakia. Increased macrophage infiltration, particularly by M2 macrophages, has been reported in oral leukoplakia undergoing malignant transformation into OSCC [32]. Moreover, a reciprocal interaction between regulatory T cells and M2 macrophages has been observed, leading to the suppression of anti-tumor immune responses [33]. These findings suggest that M2 macrophages, characterized by their roles in tissue remodeling and immunosuppression, actively contribute to tumorigenesis and the establishment of an immunosuppressive microenvironment [34].

Recent insights also highlight the role of cancer stem cells (CSCs) in orchestrating macrophage behavior within dysplastic tissues. CSCs localized in the basal layers of epithelial dysplasia have been shown to promote M2 polarization through the secretion of vascular endothelial growth factor (VEGF), thereby contributing to both macrophage modulation and angiogenesis [35]. This bidirectional communication between CSCs and tumor-associated macrophages (TAMs) is thought to be a critical driver of the transition from dysplasia to malignancy.

## 6. Tumor Invasion

**Figure 3:**
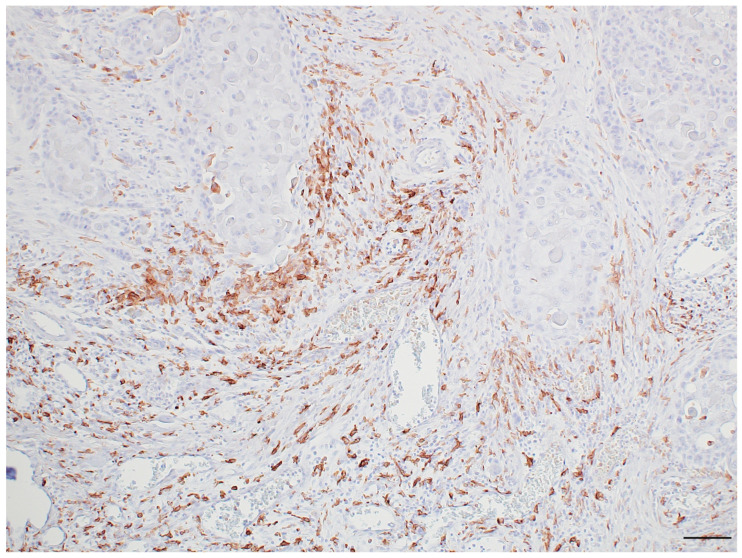
Immunohistochemical staining image showing macrophage aggregation in the infiltration area of OSCC. CD163-positive macrophages accumulate and adhere to the tumor nests, forming a dendritic morphology in the affected area. Scale bar; 100 μm. Anti-CD163 antibody, abcam, ab182422, 1:200.

TAMs are known to interact with cancer cells to promote tumor invasion. Our recent histological study showed macrophage aggregation in the infiltration area of OSCC, with CD163-positive macrophages accumulating and adhering to the tumor nests, forming a dendritic morphology in the affected area (Figure 3). Macrophages are actively recruited to the invasive front of tumors through chemotactic cues secreted by cancer cells, where they contribute significantly to facilitating tumor infiltration and progression [36,37]. After reaching the front of the tumor, TAMs secrete epidermal growth factor (EGF) and other migration-promoting factors that promote cancer cell motility and extracellular matrix remodeling to facilitate tumor progression [38,39].

It has been reported that CSF-1 plays an important role in the invasion of TAMs into tissues. Specifically, CSF-1 stimulates the survival, proliferation, and differentiation of monocytes and promotes the proliferation and motility of macrophages in breast cancer [40]. In breast cancer tissues from MMTV-PyMT mice, a developmental model of breast cancer, CSF-1 expression by cancer cells leads to TAM infiltration via CSF-1 receptor signaling. Furthermore, EGF produced by TAMs in tumor tissues activates EGF receptors expressed on breast cancer cells, establishing a positive feedback loop between cancer cells and TAMs through CSF-1/EGF signaling [40]. In a pancreatic cancer xenograft mouse model, blockade of CSF-1 signaling reduced the number of TAMs that congregated in the tumor tissue and inhibited tumor growth, indicating that CSF-1 expression correlates with TAM invasion [41]. Additionally, other chemoattractants, such as CXCL12 and heregulin β1 (HRGβ1), appear to utilize a similar EGF-dependent mechanism such as the paracrine loop [42]. In OSCC, CSF-1 is involved in TAM-mediated tumor invasion. Guo et al. showed that elevated CSF-1 expression of cancer cells correlates with increased TAM infiltration into tumor tissues [43]. Immunohistochemical and flow cytometric analyses have also shown that TAMs express EGF in OSCC, suggesting that EGF expressed by these TAMs may promote OSCC invasion [44]. M2-like macrophages secrete EGF, which increases the motility of HNSCC cells by enhancing invadopodia formation. In addition, Gao et al. reported that head and neck squamous cell carcinoma (HNSCC) cells promote the differentiation of monocytes into M2-like macrophages through the secretion of C-C motif chemokine ligand 2 (CCL2, also known as MCP-1). These subcellular protrusions contribute to extracellular matrix degradation, thereby enhancing both local invasion and distant dissemination of tumor cells. Additionally, epidermal growth factor (EGF) stimulates the expression of CCL2 in HNSCC cells, promoting monocyte recruitment and their polarization toward an M2-like phenotype, ultimately establishing a reinforcing paracrine feedback loop [45].

In the TME, TAMs contribute to tumor progression through various immunosuppressive mechanisms. They secrete cytokines such as IL-10 and transforming growth factor-β (TGF-β), which induce M2 macrophage polarization and suppress T cell-mediated immune responses by promoting regulatory T cell induction and inhibiting Th1/Th2 helper T cells [46,47]. TAMs also inhibit cytotoxic CD8^+^ T cells and NK cells via secretion of IL-10, TGF-β, and PEG2 [48,49] and overexpress CTLA-4, further dampening TCR signaling [50].

In nasopharyngeal carcinoma, glioblastoma, and hepatocellular carcinoma, increased PD-L1 expression has been detected in monocytes, indicating systemic immune modulation by macrophage-lineage cells [51,52,53]. Additionally, TAMs express immune checkpoint molecules such as PD-L1 and PD-L2, further contributing to immune evasion. CD163- and CD204-positive TAMs have been reported to promote OSCC infiltration via IL-10 and PD-L1 production [54], while CD68- and CD163-positive TAMs directly suppress activated T cells through PD-L1–PD-1 interaction, leading to T cell apoptosis and immune escape [55]. Kuang et al. demonstrated that PD-L1 expression in TAMs is regulated by the JAK2/STAT3 signaling pathway activated by OSCC-derived GM-CSF, highlighting a tumor-intrinsic mechanism enhancing immune suppression [56]. Collectively, these findings suggest that TAMs establish an immunosuppressive TME in OSCC via both cytokine-mediated and immune checkpoint-dependent mechanisms, thereby promoting tumor progression and immune evasion.

Beyond secreted factors, TAMs also modulate tumor behavior via exosome-mediated communication. Exosomes released by TAMs can alter OSCC cell phenotypes; co-culture with exosome-primed macrophages enhanced OSCC cell migration ability [22]. M2-derived exosomal miR-23a-3p has been implicated in promoting OSCC progression. In addition, miR-21-5p, an extracellular vesicle derived from TAMs, migrates to endothelial cells and targets LATS1 and VHL mRNA in endothelial cells, inhibiting YAP1 phosphorylation, subsequently enhancing YAP1-mediated HIF-1α transcription and reducing VHL-mediated HIF-1α ubiquitination [57]. Similarly, M2 macrophage-derived exosomal miR- 31-5p can make tumor suppressor LATS2 gene inhibited and facilitate the progression of OSCC by inhibiting the Hippo signaling pathway [58]. Exosomes released by TAMs, particularly those containing high levels of ANXA3, have been implicated in enhancing resistance to ferroptosis in laryngeal squamous cell carcinoma cells, thereby promoting their invasive potential [59]. Taken together, the abovementioned studies investigating the role of TAMs in OSCC suggest a critical contribution of TAMs to OSCC invasion (Table 1).

Recent studies have highlighted the critical role of microbial biofilm niches in shaping the tumor immune microenvironment of oral squamous cell carcinoma (OSCC). CD163, a representative marker of M2 macrophages, recognizes and binds bacterial elements derived from both Gram-positive and Gram-negative species, leading to enhanced production of pro-inflammatory cytokines in monocytes [60]. The detection of bacterial populations within tumor tissues has been associated with shifts in local inflammatory responses and unfavorable clinical prognoses [61]. Fusobacterium nucleatum (Fn)*,* a key constituent of oral biofilms, has been shown to induce M2-like macrophage polarization through a CXCL2-mediated feedback loop between tumor cells and macrophages. This interaction promotes an immunosuppressive phenotype that enhances OSCC proliferation, invasion, and metastasis [62]. And Fn outer membrane vesicle-mediated lactate elevation via GLUT1 was shown to promote an immunosuppressive, M2-like TAM phenotype that accelerates OSCC progression [63]. In contrast, Neuzillet et al. demonstrated that intratumoral Fn correlates with altered immune-related gene expression and poor prognosis, consistent with its ability to induce an M2-skewed TAM phenotype in the OSCC microenvironment [64].

Additionally, Wei et al. reported that periodontitis-associated Porphyromonas activate γδT cells, which are necessary for the IL-17/signal transducer and activator of the STAT3 pathway, promote M2 macrophage infiltration, and drive chronic inflammation and tissue remodeling conducive to invasion in OSCC [65]. Porphyromonas gingivalis within oral biofilms activates TAMs via DOK3 signaling, facilitating immune evasion and OSCC recurrence [66].

Taken together, these studies converge on a model in which microbial biofilm niches program TAMs to the M2 phenotype by secreting metabolites, outer membrane vesicles, and surface molecules to enhance immunosuppression, tumor cell invasion, and recurrence in OSCC.

## 7. TAMs as Biomarkers

As it has become clear that TAMs contribute to tumor progression, their utility as biomarkers is attracting attention. Specifically, tumor invasion by TAMs has been reported to be a poor prognostic factor in many malignancies, including oral cancer [67,68,69]. Currently, CD68, CD163, CD206, and CD204 are the most commonly used TAM markers for immunostaining. Moreover, IHC analysis of the pan macrophage marker CD68 and the M2-like marker CD163 has demonstrated their prognostic utility in OS; CD163 was a stronger prognosticator, as indicated by multivariate meta-analysis. CD163-positive TAMs also correlate with disease-free survival and progression-free survival, outcomes that are more relevant to patients, thus showing promising results for future clinical implementation [70,71].

With recent developments in transcriptome analysis, analysis using specific gene signatures of TAMs has also been reported in head and neck, breast, and other cancers [72,73,74]. The importance of TAMs as biomarkers will increase further when novel immunotherapies targeting TAMs are put to practical use. Furthermore, the presence of TAM-like cells circulating not only locally in the tumor tissue but also in the peripheral blood has been reported and is being investigated as a new biomarker because it is easy to collect samples [75].

## 8. Therapeutic Strategies Targeting TAMs

TAMs are potential targets for combination therapy in cancer treatment [15]. Several studies have recently proposed targeting the TAM pathway to prevent cancer development; signaling pathways such as NF-κB and cytokines released in the tumor microenvironment through the interaction between OSCC cells and TAMs are attractive targets [76,77]. Inhibitors of cytokines involved in tumor signaling pathways could be used to combat cytokines involved in promoting the malignant cycle, particularly between OSCC cells and TAMs. Given that disease-free and overall survival rates for OSCC patients have remained unchanged over the past 30 years, new treatment options are urgently needed. Below, we describe therapies targeting TAMs that are beginning to be applied outside of OSCC. However, since NF-κB is involved in various cellular functions such as inflammatory response, immune response, cell survival, and apoptosis inhibition, it may act not only on TAMs but also on normal immune cells and epithelial cells, and systemic inhibition may result in serious side effects such as immunosuppression and increased risk of infection. In addition, cytokine signals (e.g., IL-10, TGF-β, IL-6, etc.) form a complementary and redundant network, and inhibition of one may result in compensatory activation of other pathways. Therefore, inhibition of a single cytokine may have a limited effect.

Several clinical trials targeting TAMs are ongoing, evaluating agents such as CSF1R inhibitors and CCR2 antagonists in solid tumors, including head and neck cancers. Although few OSCC-specific trials have been completed, these studies highlight the potential of TAM-targeted strategies in this malignancy. Combining TAM-targeted therapies with conventional treatments (e.g., chemotherapy, radiotherapy) or immune checkpoint inhibitors may further enhance therapeutic efficacy.

As mentioned above, TAMs have recently been thought to have two distinct origins, bone marrow and yolk sac, and many approaches have been explored to prevent the migration of bone marrow-derived monocytes into tissues [6,7]. Although many factors are involved in monocyte migration as described above, the interaction between CSF1 and CSF1R plays a central role in monocyte migration and maturation of TAMs, and it has been reported that treatment targeting CSF1 and CSF1R inhibits the infiltration of TAMs, especially M2-like populations in mouse models [78]. The CSF1R inhibitor pexidartinib was reported in a model of malignant melanoma to eliminate TAMs and induce differentiation into M1-like macrophages, leading to an increase in and activation of tumor-infiltrating T cells. BLZ945 and ARRY-382, which are also CSF1R inhibitors, have also been reported to remove TAMs and reduce tumor-promoting effects [79,80]. On the other hand, emactuzumab, an anti-CSF1R antibody, showed a TAM-eliminating and T-cell-infiltrating effect in a mouse model of colorectal cancer, while emactuzumab administration to humans showed a decrease in TAM and an increase in CD8/CD4 ratio [81].

TAM’s own phagocytosis is inhibited by cancer cells. Signal regulatory protein alpha (SIRPα), a membrane protein expressed on macrophages, is known as a “don’t eat me” signal, as it inhibits phagocytosis when bound by cells expressing the ligand CD47. CD47 is abundantly expressed on erythrocytes, platelets, and neurons, but cancer cells also express CD47 and evade phagocytosis by macrophages [82,83]. CD47 is also expressed in head and neck cancer and is a poor prognostic factor. Therefore, approaches using anti-CD47 or anti-SIRPα antibodies to induce phagocytosis of cancer cells by TAMs have been attempted, and it has been reported that blocking human head and neck cancer cells with CD47 and anti-CD47 antibodies increases phagocytosis by macrophages [84]. Table 2 shows the summary of the above researches.

## 9. Conclusions

TAMs contribute to tumor growth and progression through various mechanisms and are attracting attention as new biomarkers and therapeutic target cells. Further elucidation of their origin and function is expected to make TAMs a new option for cancer immunotherapy, along with immune checkpoints, in various malignant tumors, including those in the oral cavity.

## Figures and Tables

**Table 1:** Studies targeting TAMs in OSCC progression.

Study (Author, Year, Reference)	Mechanism of Action	Key Factors	Effects on OSCC
Pollard et al. (2009) [36], Condeelis et al. (2006) [37]	Recruitment of macrophages to tumor invasion front	Chemotactic factors secreted by cancer cells	Promotes TAM accumulation at tumor front, enhancing invasion
O’Sullivan et al. (1993) [38], Singh et al. (2010) [39]	TAMs secrete migration-promoting factors	EGF, ECM remodeling factors	Enhances cancer cell motility and extracellular matrix degradation
Goswami et al. (2005) [40]	CSF-1 stimulation of monocyte survival, proliferation, and differentiation	CSF-1, CSF-1R	Promotes TAM invasion into tissues and enhances tumor invasion
Hernandez et al. (2009) [42]	Paracrine signaling loop between TAMs and cancer cells	CXCL12, HRGβ1	Drives tumor cell migration and invasion
Li et al. (2020) [41]	CSF-1 blockade reduces TAM infiltration	CSF-1	Inhibits tumor growth in a pancreatic cancer model
Guo et al. (2020) [43]	High CSF-1 expression in OSCC cells enhances TAM invasion	CSF-1	Correlates with TAM infiltration and OSCC progression
Haque et al. (2019) [44]	EGF expression in TAMs promotes OSCC invasion	EGF	Enhances OSCC cell motility and invasion
Gao et al. (2016) [45]	TAMs induce invadopodia formation via EGF	EGF, CCL2 (MCP-1)	Increases ECM degradation and tumor invasion
Ng et al. (2013) [46], Savage et al. (2008) [47]	TAMs suppress T cell immune responses	IL-10, TGF-β	Induces regulatory T cells and inhibits Th1/Th2 responses
Wang et al. (2023) [48] Lyford-Pike et al. (2013) [49],	TAMs suppress CD8+ T cells and NK cells	IL-10, TGF-β, PEG2	Enhances tumor immune evasion
Takahashi et al. (2019) [52], Bloch et al. (2013) [53], Kuang et al. (2009) [56]	Increased PD-L1 expression in monocytes/macrophages	PD-L1	Suppresses anti-tumor immunity in OSCC and other cancers
Kubota et al. (2017) [54]	CD163+CD204+ TAMs regulate T cells	IL-10, PD-L1	Promotes immune evasion of OSCC cells
Hartley et al. (2018) [55], Suárez-Sánchez et al. (2020) [51]	TAMs overexpress immune checkpoint molecules	PD-L1, CTLA-4	Inhibits TCR signaling and promotes T cell apoptosis
Wang et al. (2023) [48]	JAK2/STAT3 pathway regulates PD-L1 expression	GM-CSF, JAK2/STAT3	Promotes immune escape of OSCC cells
Yan et al. (2024) [57]	Exosome-mediated OSCC progression	miR-23a-3p, miR-21-5p	Enhances OSCC cell migration and angiogenesis
Yuan et al. (2021) [58]	M2 macrophage-derived exosomal miR-31-5p inhibits tumor suppressors	miR-31-5p, LATS2	Promotes OSCC progression by inhibiting Hippo signaling pathway

**Table 2:** Therapeutic strategies targeting TAMs.

Drug/Antibody (Reference)	Target	Mechanism of Action	Clinical Trials/Findings
Pexidartinib [78]	CSF1R	Inhibits CSF1R signaling to deplete M2-like TAMs →Promotes M1 polarization and T cell activation	In melanoma models: TAM depletion and T cell infiltration reported
BLZ945 [79]	CSF1R	Depletes TAMs and suppresses tumor-promoting effects	Reported tumor reduction in preclinical studies
ARRY-382 [80]	CSF1R	Depletes TAMs and suppresses tumor-promoting effects	Preclinical efficacy demonstrated
Emactuzumab [81]	CSF1R (antibody)	Eliminates TAMs and enhances immune activation via increased CD8/CD4 T cell ratio	In colorectal cancer model: TAM reduction In humans: increased CD8/CD4 ratio
Anti-CD47 antibody [82,83]	CD47	Blocks “don’t eat me” signal on cancer cells →Promotes TAM-mediated phagocytosis	CD47 expression is a poor prognostic marker in HNSCC Anti-CD47 increases phagocytosis
Anti-SIRPα antibody [84]	SIRPα	Inhibits CD47-SIRPα interaction →Restores macrophage phagocytic function	Early-stage or preclinical reports available
